# Deep Ultraviolet Laser Ablation Electrospray Ion Mobility Mass Spectrometry

**DOI:** 10.1021/jasms.5c00403

**Published:** 2026-01-21

**Authors:** Kelcey B. Hines, Neda Feizi, Touradj Solouki, Kermit K. Murray

**Affiliations:** † Department of Chemistry, 5779 Louisiana State University, Baton Rouge, Louisiana 70803, United States; ‡ Department of Chemistry and Biochemistry, 14643 Baylor University, Waco, Texas 76798, United States

## Abstract

A solid-state deep ultraviolet (DUV) optical parametric oscillator (OPO) at 206 nm wavelength was utilized for laser ablation electrospray postionization of peptides and proteins coupled with ion mobility mass spectrometry (IM-MS) analysis. Peptide and protein standard solutions were spray deposited on quartz microscope slides to obtain thin, surface-coated dry films. The pulsed laser irradiated the solid sample biomolecules in transmission geometry, and the ablated surface material merged with the electrospray plume for ionization before entering the IM-MS instrument. Laser ablated biomolecules remained intact, and no fragmentation was observed from the peptide or protein standards. The efficiency of ionization was estimated at approximately 1% for instantaneous ion signal; however, the signal was not stable over time. Mass spectra of laser ablation electrospray for peptide and protein standards revealed multiply charged ions similar to those observed in direct electrospray ionization (ESI) MS. The ion mobility drift times for proteins from laser ablation electrospray (LA-ESI) experiments were indistinguishable from those observed in direct ESI.

## Graphical abstract

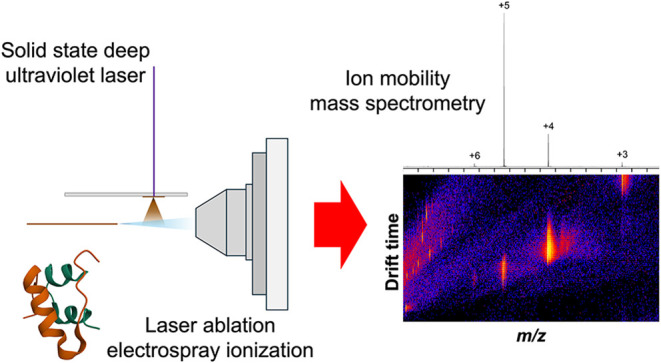

## Introduction

Since its introduction 2 decades ago,^1^ ambient mass spectrometry (MS) has evolved into a versatile suite of ionization methods for sampling surfaces with analysis by mass spectrometry.^2,3^ Surface sampling and analysis by MS can be accomplished using charged droplets for electrospray ionization (ESI),^1^ via surface sampling probe,^4^ or electrical discharge plasma.^5,6^ Lasers can be used to remove analytes from surfaces for subsequent ionization of the ablated material via plasma or ESI.^7,8^ Lasers are advantageous for ambient MS because they do not require solvent extraction or surface contact and can be focused to a small spot which facilitates localized sampling and imaging.^9^

Ambient laser ablation (LA) ionization is typically accomplished by merging the ablation plume with an ESI source to generate ions.^10,11^ Pulsed nanosecond ultraviolet 337 nm nitrogen lasers were used initially,^8,12^ but it was subsequently discovered that 3 μm wavelength nanosecond mid-infrared lasers can utilize the vibrational absorption of residual water in the sample to facilitate efficient ablation.^13–15^ Ultrafast picosecond and femtosecond lasers have been used in LA-ESI for IR and nonresonant absorption.^16–18^ The nomenclature and acronyms associated with laser ablation and electrospray are numerous; here, we use the hyphenated acronym LA-ESI to refer to any technique in which a pulsed laser ablates material, generating a plume that merges with an electrospray to form analyte ions.

We recently demonstrated LA-ESI using deep ultraviolet (DUV) 193 nm excimer laser for LA-ESI.^19^ Although sufficiently energetic to fragment gas phase ions,^20^ this wavelength does not produce extensive fragmentation of laser ablated biomolecules in LA-ESI^19^ or laser ablation capture for bottom-up proteomics of tissue.^21,22^

DUV lasers have several potential advantages for ambient surface sampling. They are well-known for their ability to cleanly and precisely cut tissue due to their low optical penetration depth and the ability to photochemically break tissue bonds.^23^ Furthermore, DUV lasers can be focused to a smaller diffraction limited spot size compared to IR lasers if the beam quality is comparable.^24^ Additionally, the low optical penetration depth in tissue promotes the formation of small ablation particles from which biomolecules can be efficiently extracted.

Although 193 nm ArF excimer lasers have been used for laser ablation sampling and ionization, they have the disadvantages of being relatively large and requiring hazardous fluorine gas for their operation, making adaptation to an ambient ion source difficult. Additionally, excimer lasers have significantly lower beam quality compared to solid-state lasers, which is not well-suited to tight focusing and spatially precise surface sampling.

In this work, we describe the use of a DUV solid-state laser system comprising a Nd:YAG pumped optical parametric oscillator (OPO) for laser ablation with electrospray postionization for ion mobility MS (IM-MS). Peptide and protein samples were spray deposited on quartz microscope slides and positioned a few millimeters above the ESI source of the ion mobility mass spectrometer. The biomolecule deposits were ablated at 206 nm in transmission mode and entrained in the electrospray, and the resulting ions were sampled into the mass spectrometer. The mass spectra and ion mobility drift plots were acquired for ions formed by LA-ESI and compared with those from direct ESI.

## Materials and Methods

The DUV LA-ESI ion source was constructed by using an OPO laser system and the modified electrospray ion source of a commercial mass spectrometer. The DUV laser is a Nd:YAG pumped OPO (Opotek, Carlsbad, CA) that is tunable from 193 to 210 nm with a 6 ns pulse width and a maximum repetition rate of 20 Hz. The laser has a pulse energy that ranges from 100 μJ at 193 nm to 800 μJ at 210 nm. The maximum pulse energy is obtained at the longer wavelength end of the tuning curve; it was found that 206 nm was the shortest wavelength that consistently provided a high pulse energy and was therefore selected for the experiments described below.

The laser was mounted on a 1.3 m tall table constructed from 25 mm optical rails and two 24 in. square aluminum optical breadboards. The laser energy was adjusted using a home-built counter-rotating Brewster attenuator with a set of 1 mm thick 25 × 15 mm^2^ quartz optical flats, and the beam was directed downward to the target microscope slide with a UV fused silica right angle prism. The attenuated beam was focused with a 25 mm diameter and 10 mm focal length CaF_2_ lens. For the ablation experiments, the laser was slightly defocused to a 200 μm spot, as measured using laser burn paper. It was found that a larger spot size was necessary in the current configuration to obtain an adequate ion signal. The laser energy was measured using a pyroelectric laser energy detector (QE12LP-S-MB-D0, Gentec, Quebec City, Canada).

A schematic of the DUV LA-ESI ion source is shown in Figure 1. The ablation target was a quartz microscope slide mounted above the electrospray, with the short axis of the slide parallel to the spray axis. Samples were ablated from the downward-facing side of the slide in transmission geometry, with the material ablated from the bottom into the electrospray. Although reflection mode ablation was used previously for 193 nm DUV LA-ESI,^19^ transmission mode was chosen for compatibility with the ion source. The bottom of the microscope slide was 5 mm above the electrospray plume axis. The electrospray emitter was a fused silica capillary (Polymicro Technologies, Phoenix, AZ) with a 170 μm inner diameter and a 350 μm outer diameter. The 30 mm long capillary was held using a polyetheretherketone (PEEK) reducing sleeve in a 1/16 in. stainless steel union to which the electrospray was applied. The ESI tip to skimmer distance was 15 mm and intersected the axis of the laser beam 5–10 mm downstream of the capillary tip.

**Figure 1:**
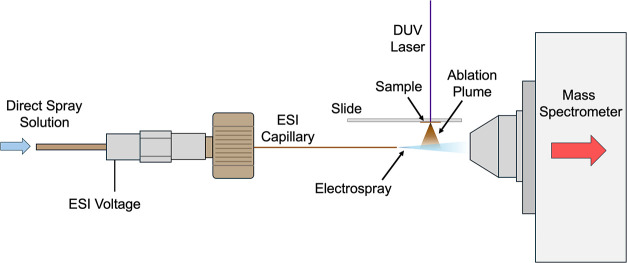
Laser ablation electrospray ion source comprising an electrospray capillary, sample on a quartz microscope slide irradiated in transmission mode with a 206 nm OPO, and mass spectrometer source inlet.

The sample target was translated with respect to the laser and spray by using a three axis mechanical stage. The translation stage comprised one 46 mm linear stage (Model 433, Newport, Irvine, CA) parallel to the axis of the electrospray and two 25 mm stages (Model 423, Newport) for perpendicular horizontal and vertical motion. The two horizontal axes were driven by DC servo motorized actuators (LTA-HS, Newport) and motion controllers (SMC100CC, Newport) operated via a custom LabView program (National Instruments, Austin, TX); the vertical axis was controlled manually with a micrometer. Slides were affixed with double-sided tape to a 25 mm aluminum construction cube (CC-1, Newport) that was attached to the stage.

Samples were produced using the peptide bradykinin and the proteins bovine serum albumin (BSA) and human insulin (Sigma-Aldrich, St. Louis, MO). Each biomolecule sample was dissolved in a 1:1 (v/v) mixture of deionized water, purified to resistivity of 18.2 MΩ at an ambient temperature of approximately 22.0 ± 0.2 °C using an in-house ultrapurification system (Millipore Sigma, St. Louis, MO), and acetonitrile (≥99.9%; Fisher-Scientific) at 1 mg/mL concentration for bradykinin and 2 mg/mL for insulin and BSA. The analyte solution was spray deposited on the microscope slide using a gravity feed airbrush (Model G22, TCP Global, Las Vegas, NV) at 207 kPa air pressure at a surface distance of 5 cm using 8 passes with 30 s dry time between cycles. The spot size was approximately 1 × 2 cm^2^, with 1 mL consumed for each deposit. Assuming a uniform deposit, the sample thickness was approximately 10 μm, corresponding to approximately 1 mg/cm^2^ analyte.

The electrospray solvent comprised a 1:1 (v/v) mixture of high-pressure liquid chromatography (HPLC)-grade water and acetonitrile with 0.1% formic acid (≥98%, Sigma-Aldrich), which was delivered to the capillary at a flow rate of 1 μL/min with a syringe pump. A potential between 2.3 and 2.9 kV was applied to the emitter, which provided a stable spray with minimal nozzle-skimmer fragmentation. For direct infusion, standards were dissolved in an electrospray solvent to prepare a 1 μM solution.

For laser ablation electrospray, the laser was fired at a repetition rate of 20 Hz, and the sample slide was moved in a serpentine fashion at a velocity of 1 mm/s with 2 mm lines spaced by 0.1 mm to provide a quasi-continuous source of ablated analyte. Alignment of the laser with the electrospray axis was critical, and under optimized conditions, experiments could be conducted for several minutes, encompassing multiple 2–4 s duration signals in one run. Accounting for the approximately 80% transmission through the quartz microscope slide, approximately 300 μJ/pulse was delivered to the sample, which corresponds to an average laser fluence of 9 kJ/m^2^ for the Gaussian profile beam.

Mass spectra and ion mobility data were obtained in positive-ion mode on a Synapt G2-S traveling wave ion mobility quadrupole time-of-flight spectrometer (TWIMS-q-ToF) IM-MS system. MassLynx software V4.2 (Waters, Milford, MA) was used for data acquisition and instrument control. All experiments were conducted with four replicates for each analyte; the experimental parameters were optimized to achieve the best signal-to-noise ratio. The optimized pre-IM trap collision voltages were set to 5 V for bradykinin and 10 V for insulin and BSA. For bradykinin and insulin ion mobility measurements, the gas flow rates in the Triwave cell were set to 5 mL/min argon, 110 mL/min helium cell, and 95 mL/min IMS cell. Optimized IM parameters for BSA were 5 mL/min trap gas, 110 mL/min helium cell, and 45 mL/min IMS cell gas flows.

ESI mass spectra were acquired over 2.4 s for bradykinin and insulin and 3 s for BSA. Assuming complete spot-by-spot removal, the total analyte quantity ablated to obtain a mass spectrum was 2 nmol for bradykinin, 840 pmol for insulin, and 90 pmol for BSA. Mass spectra and ion mobility raw data were extracted from MassLynx v4.2 (Waters) and imported to OriginPro V2024b (OriginLab, Northampton, MA). The raw data was normalized to unity prior to generating mass spectra plots. Data reduction of the BSA mass spectra was achieved by averaging 50 consecutive data points. The peak area for each charge state was used to calculate the average charge states of insulin and BSA. Ion mobility and mass spectra data were used to generate drift time vs *m*/*z* plots (drift-scope plots) with DriftScope v4.2 (Waters).

## Results and Discussion

The DUV LA-ESI ion source was demonstrated on a quadrupole time-of-flight mass spectrometer equipped with ion mobility separation using peptide and protein standards. Figure 2 shows representative positive-ion mode ESI mass spectra of bradykinin (*m*/*z* range of 250–800) from (a) direct ESI and (b) DUV LA-ESI. The doubly protonated peptide [M + 2H]^2+^ at *m*/*z* 530.8 is the most intense peak observed in both mass spectra in Figure 2. A small peak, typically between 1 and 4% of the intensity of the doubly protonated peptide peak, corresponds to the singly protonated [M + H]^+^ peptide at *m*/*z* 1060.6 (expanded regions of the mass spectra are shown in Figure S1). Both mass spectra in Figure 2 contained b and y peptide fragment peaks, likely from nozzle-skimmer dissociation; their summed integrated intensity was approximately 6–20% of the [M + 2H]^2+^ peak between replicates. In the LA-ESI mass spectrum Figure 2b, a peak corresponding to the doubly protonated oxidized peptide [M + O + 2H]^2+^ at *m*/*z* 538.9 was observed at approximately 4% of the intensity of the [M + 2H]^2+^ peak. Under all conditions used in this work, direct ESI (Figure 2a) and LA-ESI (Figure 2b) yielded comparable mass spectra (Figure S1), with the most abundant ion in all cases corresponding to the doubly charged peptide.

**Figure 2:**
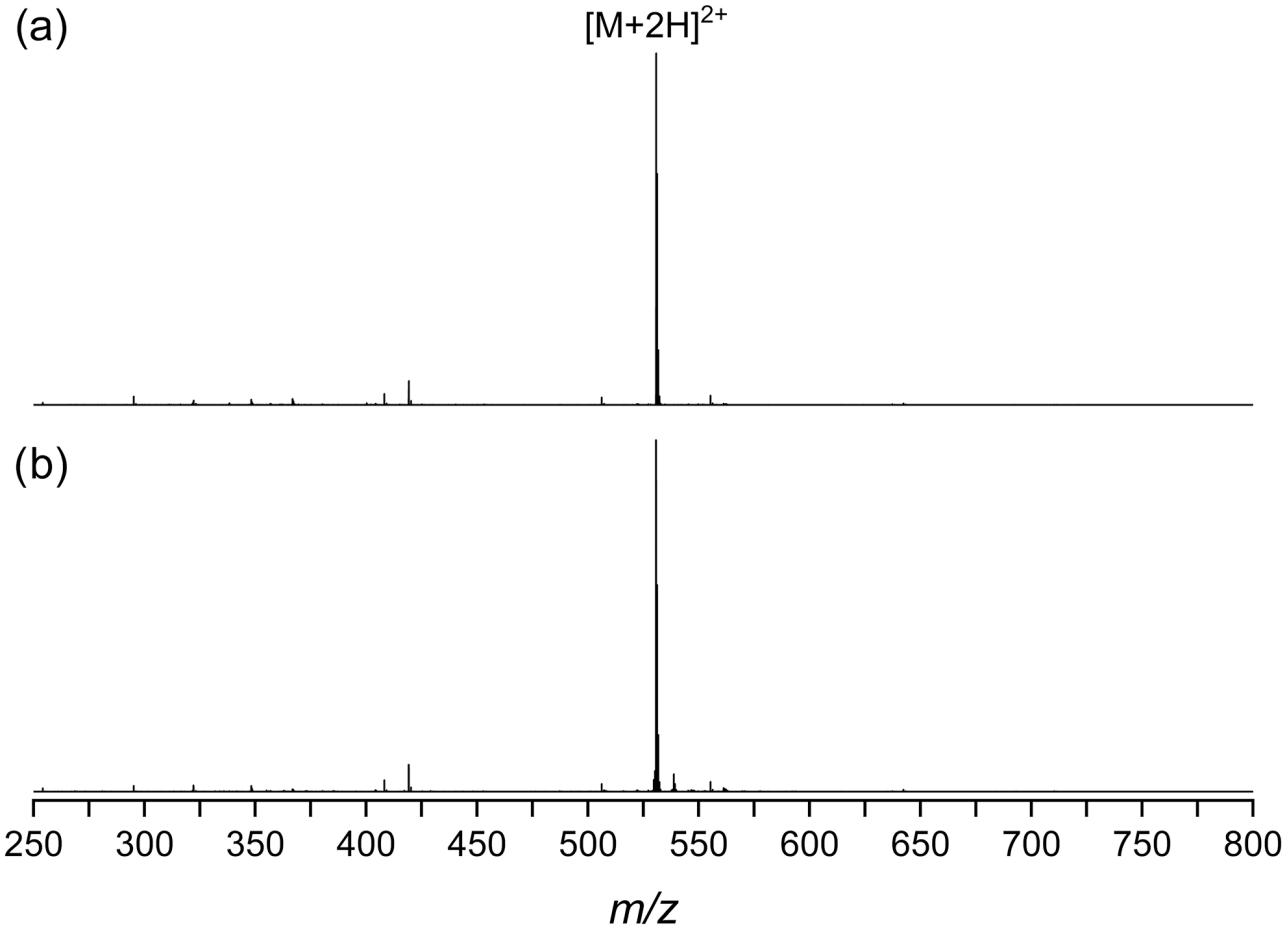
Positive-ion mode mass spectra of bradykinin acquired with (a) direct ESI and (b) DUV LA-ESI.

Figure 3 shows representative positive-ion mode mass spectra of insulin (*m*/*z* 500–2200) from direct ESI (Figure 3a) and DUV LA-ESI (Figure 3c). Corresponding drift-scope plots for direct and LA-ESI are shown in Figure 3b,d, respectively. Both direct ESI and DUV LA-ESI mass spectra show insulin charge states +3 to +6 with charge state +5 being the most abundant in both mass spectra. At a 95% confidence interval (*n* = 4), the average charge state was 4.73 ± 0.03 for direct ESI and 4.74 ± 0.02 for DUV LA-ESI.

**Figure 3:**
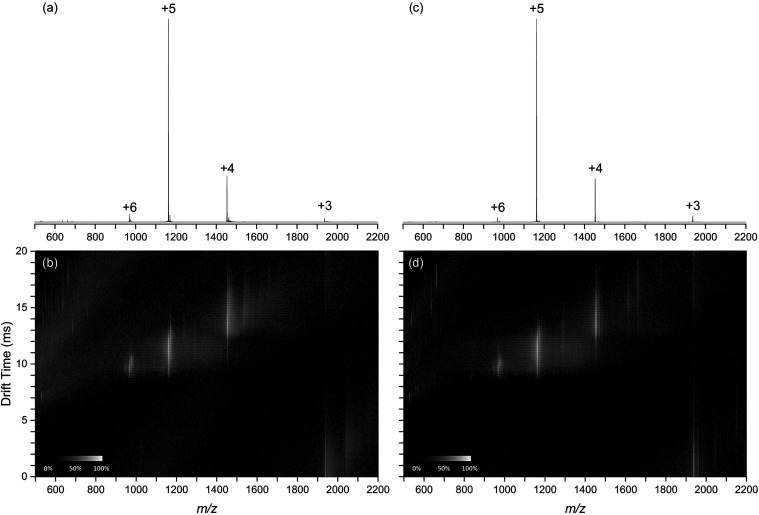
Positive-ion mode mass spectra (a, c) and drift-scope plots (b, d) of insulin acquired with direct ESI (a, b) and DUV LA-ESI (c, d).

The drift-scope plots for direct ESI (Figure 3b) and LA-ESI of insulin (Figure 3d) show four regions corresponding to ion charge states: the protonated insulin molecule and associated adduct peaks at ∼1000 *m*/*z* and 10 ms drift time for the +6 charge state, ∼1200 *m*/*z* and 11 ms for the +5 charge state, ∼1500 *m*/*z*, 14 ms for the +4 charge state, and ∼2000 *m*/*z* and 21 ms for the +3 charge state. The two less intense regions from 500 to 1000 *m*/*z* at drift times between 7 and 20 ms and from 500 to 1200 *m*/*z* at drift times from 8 to 20 ms correspond to solvent cluster ions. A drift scope plot for a solvent-blank is shown in Figure S2.

Transfer efficiency was determined using the peptide leucine enkephalin: an ablated sample was compared to an electrospray of a known quantity. Mass spectra of the leucine enkephalin peptide from a signal laser shot of DUV LA-ESI and from direct ESI integrated for a comparable time period are shown in Figure S3. The leucine enkephalin sample deposit comprised 8 nmol of protein, and approximately 1 pmol of protein was removed from a single 200 μm spot with a single laser shot. The resulting signal had a duration of approximately 3 s (Figure S4) and was compared to a 3 s integration of direct ESI with a known concentration and flow rate. A 4.3 μM solution at 1 μL/min flow rate consumed a protein quantity of approximately 180 fmol and produced a signal of 4 × 10^4^. The DUV-LA-ESI signal was 3 × 10^3^ corresponding to 13 fmol detected and an efficiency of approximately 1%. It should be noted that this efficiency was difficult to obtain for long periods and represents an upper bound of the efficiency for the reported configuration.

Figure 4 depicts representative BSA positive-ion mode mass spectra (*m*/*z* range 500–3000) for direct ESI (Figure 4a) and DUV LA-ESI (Figure 4c). Corresponding drift-scope plots are shown in Figure 4b,d. BSA was observed with charge states from +32 to +62 in both mass spectra with statistically indistinguishable average charge states of 46.2 ± 0.6 for direct and 46.1 ± 2.4 for DUV LA-ESI at 95% confidence (*n* = 4). BSA drift-scope plots (Figure 4b,d) show broad and partially resolved trend lines from the BSA charge states in addition to trend lines below 1000 *m*/*z* corresponding to singly charged solvent cluster ions.

**Figure 4:**
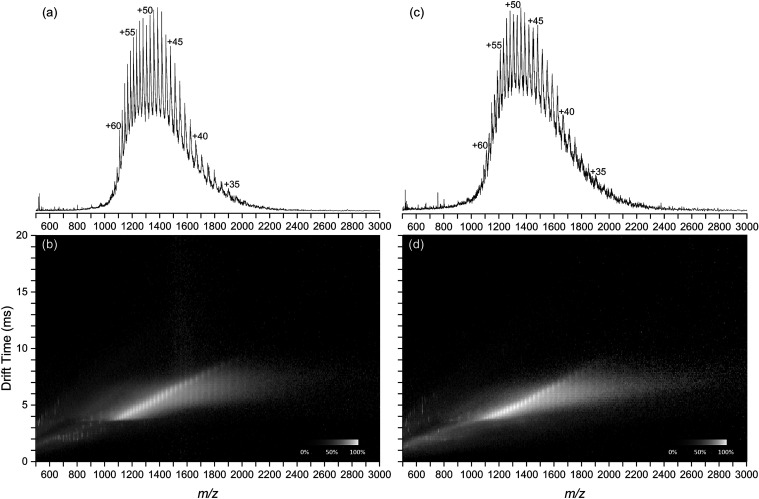
Positive-ion mode mass spectra (a, c) and drift-scope plots (b, d) of BSA acquired with direct ESI (a, b) and DUV LA-ESI (c, d).

In our previous report on DUV LA-ESI, we postulated that the energy absorber in the ablation process was either a fraction of the analyte molecules or the residual water in the sample.^19^ In the previous work, absorption of the laser energy by the stainless steel sample target was negligible because the absorption length of protein is small compared to the relatively thick protein film ablated in reflection mode.^19^ In the current study, the analyte is ablated in transmission mode, and the absorption of the laser energy by the substrate is determined by the absorption of the nominally transparent material. The quartz slide absorbs up to approximately 20% of the 206 nm laser energy passing through the material, resulting in a volumetric energy density in the substrate producing a temperature rise of at most 2 °C. This suggests that substrate heating does not play a role in ablation.

It is possible that a fraction of the analyte molecules absorb the laser energy, causing dissociation and formation of small gas phase molecules that propel intact protein into the plume in the form of small clusters and particles. This concept of a fraction of the protein molecules serving as a “sacrificial matrix” has been proposed previously to explain infrared laser desorption and ionization of intact proteins.^25^ It has been shown by laser ablation and capture that 90% of the proteins ablated by DUV ablation at 193 nm are removed intact,^21^ which suggests that at most 10% of the proteins might be completely fragmented and act as a sacrificial matrix. In our study, the protein film is approximately 10 μm thick, and the absorption depth of the 206 nm laser is approximately 1 μm. It is possible that the absorbing volume of the protein film is efficiently converted to gas-phase photochemical fragments that eject the layers above to form a material plume. The lack of observed biomolecule fragment ions suggests that an analyte absorption mechanism must involve a small fraction of the analyte ions and their fragmentation must be complete.

The absorption of energy by residual water has also been suggested as a mechanism for DUV laser energy absorption by protein thin films.^19^ The first electronic transition of water is blue-shifted and broadened from the gas to the condensed phase with a resulting maximum near 160 nm.^26–30^ The absorption is broad and is 5 orders of magnitude lower at 193 nm wavelength than at the 160 nm maximum^31,32^ and the absorption at 206 nm is approximately 5 times lower than at 193 nm.^33–35^ However, rapid heating of water by pulsed laser irradiation increases the absorption due to changes in the solvent environment on heating affecting the electronic transition energy.^29,32,36,37^ The amount of water in a protein dried deposit can be as high as 5% or more,^38,39^ depending on the protein and relative humidity, and could contribute to energy absorption. However, its contribution at 206 nm will be smaller than 193 nm or a shorter wavelength. It should be noted that the observation of oxidized bradykin peptide (Figure S1) indicates that some water photolysis occurs and this may be due to residual water absorption. Thus, while water absorption cannot be ruled out, absorption of laser energy by a sacrificial fraction of the deposited protein seems most likely.

An important aspect of the ionization mechanism is the form of the ablated protein and merging of the charged electrospray droplets. Previous studies of IR LA-ESI have shown that the optimum distance between the ablation target and ESI axis is in the range of 12–25 mm, suggesting that micrometer-scale particles with relatively long plume expansion stopping distances are responsible for transporting the analyte to the plume for droplet merging.^13,40^ In this study and our previous study,^19^ it was observed that the best ion signal was obtained with the distance between the target and the spray axis at approximately 5 mm. This result is consistent with the relatively smaller particulate obtained from UV ablation compared to IR ablation of tissue.^21^ The smaller particles have a shorter stopping distance and thus a smaller optimum target to spray distance. The smaller particle size may be advantageous for ESI droplet capture and subsequent protein solvation and ionization.

## Conclusions

A solid-state deep-UV OPO laser system was used with LA-ESI and ion mobility mass spectrometry. Ions are observed with no detectable fragmentation, but there is a limited amount of photochemical oxidation. The ionization efficiency is approximately 1%; however, acquiring a stable signal for long scans was challenging. The results are consistent with a mechanism in which laser energy absorption is likely via a small fraction of the analyte molecules in the sample, causing photochemical conversion to gas phase products that eject the remainder of the analyte into the ablation plume intact. The efficient ablation at 206 nm suggests that the Nd:YAG fifth harmonic at 213 nm may be an alternative UV source that does not require an OPO wavelength conversion.

Ion mobility of DUV laser ablated proteins was compared to the ion mobility of directly injected proteins and found to be similar. It was not possible to perform collision-induced unfolding experiments at multiple collision energies due to the limited duration of the analyte signal. Future studies will be aimed at improving the duration of the sample signal using large-area sample deposits and computer-controlled stage movement. The use of a laser ablation flow cell may allow more efficient merging of the ablated material with the ESI source. The improved system will also be used for ablation sampling from tissue and tissue imaging.

## Supplementary Material



## Notes

Positive ion mode mass spectra expanded region of bradykinin acquired with (a) direct ESI and (b) DUV LA-ESI (Supporting Figure S1); drift-scope plot of solvent blank acquired with direct ESI (Supporting Figure S2); positive-ion mode mass spectra of leucine enkephalin acquired with (a) direct ESI and (b) single shot of DUV LA-ESI (Supporting Figure S3); total ion signal of leucine enkephalin DUV LA acquired with a single laser shot (Supporting Figure S4) (PDF)
