# Supplementary material for: Deep Ultraviolet Laser Ablation Electrospray Ion Mobility Mass Spectrometry
Source: J Am Soc Mass Spectrom. 2026 Jan 21;37(2):532–8. doi: 10.1021/jasms.5c00403 (PMC12879934; doi:10.1021/jasms.5c00403)
Supplement: Supplementary file 1 [file js5c00403_si_001.pdf]

Supporting Information

Deep Ultraviolet Laser Ablation Electrospray Ion Mobility Mass Spectrometry

*Kelcey B. Hines[1], Neda Feizi[2], Touradj Solouki[2], and Kermit K. Murray[1]**

[1]Department of Chemistry, Louisiana State University, Baton Rouge, LA, 70803, USA
[2]Department of Chemistry and Biochemistry, Baylor University, Waco, TX 76798, USA

*Corresponding Author and reprint requests:

Kermit K. Murray,
331 Choppin Hall,
Department of Chemistry,
Louisiana State University,
Louisiana, 70803, United States
Phone: +1 (225) 578-3417
Fax: +1 (225) 578 3458
E-mail:kkmurray@lsu.edu

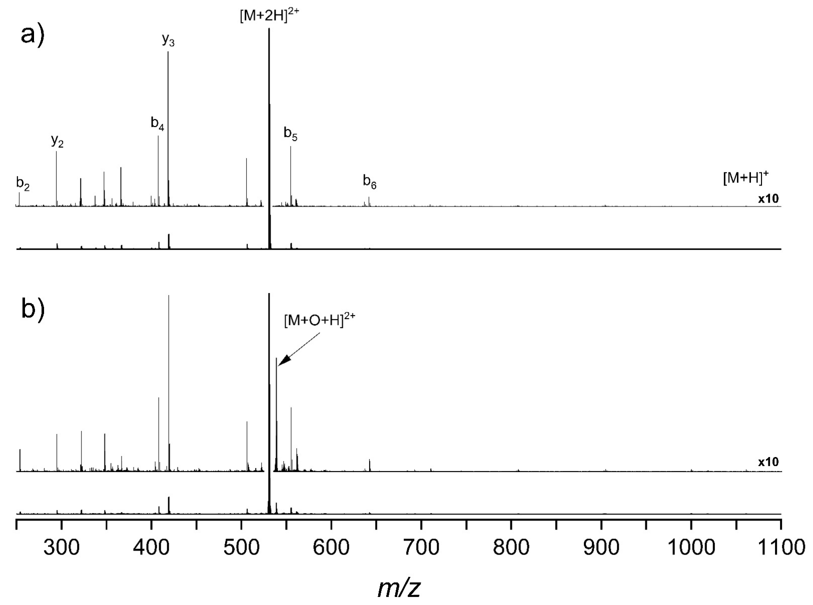

Figure S1. Positive ion mode mass spectra expanded region of bradykinin acquired with a) direct ESI and b) DUV LA-ESI.

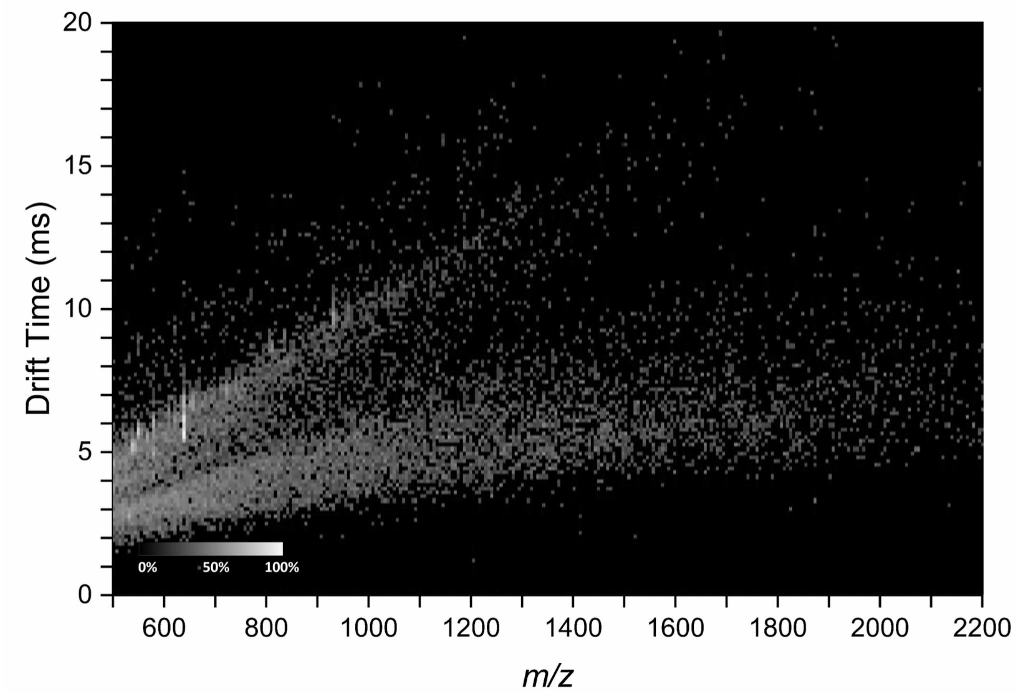

Figure S2. Drift-scope plot of solvent blank acquired with direct ESI.

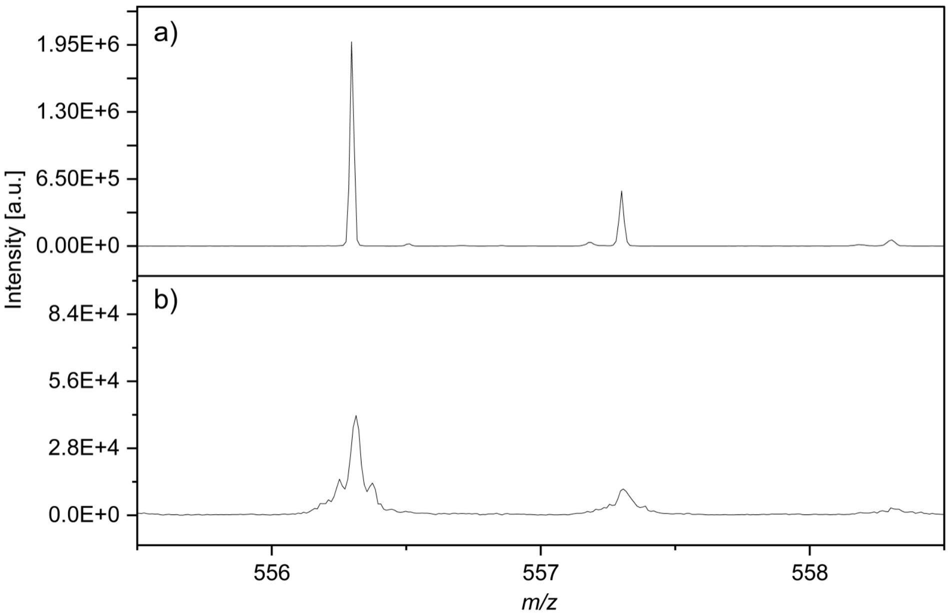

Figure S3. Positive-ion mode mass spectra of leucine enkephalin acquired with a) direct ESI and b) single shot DUV LA-ESI.

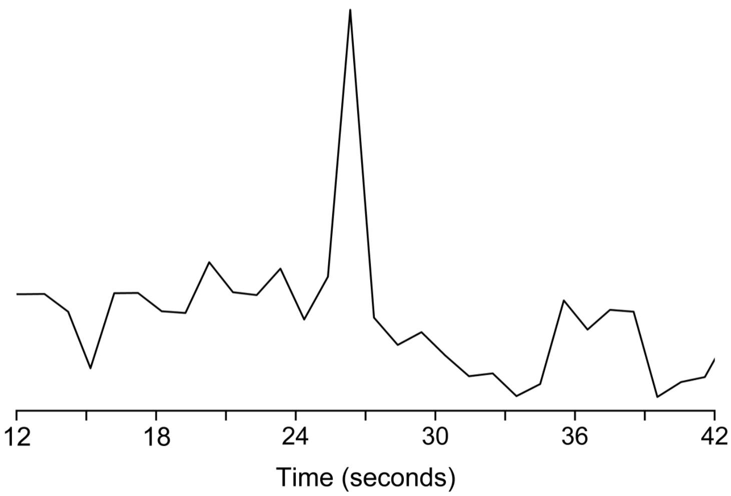

Figure S4. Total ion signal of leucine enkephalin DUV LA acquired with a single laser shot.
